# Factors associated with mortality among hospitalized patients with COVID-19 disease treated with convalescent plasma

**DOI:** 10.1128/mbio.01777-23

**Published:** 2023-11-08

**Authors:** Armando M. Perichon, Andrea Acosta, Liliana Di Tulio, Maria José Munuce, Stella Pezzotto, Oscar Bottasso, Esteban C. Nannini

**Affiliations:** 1 Centro Único de Donación, Ablación e Implante de Órganos, Ministerio de Salud, Rosario, Santa Fe, Argentina; 2 Centro Regional de Hemoterapia Sur, Ministerio de Salud, Rosario, Santa Fe, Argentina; 3 Laboratorio de Medicina Reproductiva–Área Bioquímica Clínica-Facultad de Ciencias Bioquímicas y Farmacéuticas, Universidad Nacional de Rosario, Rosario, Argentina; 4 Instituto de Inmunología Clínica y Experimental de Rosario, Universidad Nacional de Rosario-CONICET, Rosario, Argentina; 5 Consejo de Investigaciones, Universidad Nacional de Rosario, Rosario, Argentina; 6 Servicio de Infectología, Sanatorio Británico, Rosario, Santa Fe, Argentina; Johns Hopkins Bloomberg School of Public Health, Baltimore, Maryland, USA

**Keywords:** SARS-CoV-2, COVID-19, pneumonia, convalescent plasma, transfusion, immunotherapy

## Abstract

The use of convalescent plasma (CP) for hospitalized patients with SARS-CoV-2 infection might be a useful option in certain settings. Soon after the outbreak of COVID-19, the National Ministry of Health of Argentina recommended the use of CP transfusion for hospitalized patients with COVID-19 disease. Between 1 June and 3 October 2020, 480 patients, excluding those on invasive mechanical ventilation (IMV), received at least one CP infusion in the province of Santa Fe. We aimed to find factors associated with mortality among this cohort of patients. The median age was 60 years (interquartile range: 49–69 years) and 320 (66.7%) were males. Most of these patients (93.75%) received a single CP infusion, 82.1% and 95.6% before day 4 and day 7 of hospitalization, respectively. Anti-SARS-CoV-2 titers were determined in the CP units administered using Elecsys Anti-SARS-CoV-2 S assay. At 28 days of follow-up, 250 patients were discharged (52.1%), 131 (27.3%) remained hospitalized without and 16 (3.3%) with oxygen requirement, 27 (5.6%) were on IMV, and 56 (11.7%) had died. In the multivariate logistic regression analysis, the factors significantly associated with 28-day mortality were (i) requirement of IMV, (ii) the administration of CP after the third day of hospitalization, (iii) age, and (iv) number of comorbidities. The qualitative and quantitative analyses of antibodies against SARS-CoV-2 in the infused CP were not associated with mortality. Our findings may imply a seemingly favorable effect of CP administration among patients with severe COVID-19 disease when infused sooner after hospitalization.

**IMPORTANCE:**

The use of convalescent plasma (CP) could be an option for patients with severe COVID-19, especially in poor-resource countries where direct antiviral drugs are not commercially available. Currently, the U.S. Food and Drug Administration limits the CP administration for outpatients and inpatients with COVID-19 who are immunocompromised and only if high levels of anti-SARS-CoV-2 antibodies are confirmed in the CP unit. Although most of the randomized clinical trials failed to show a clear-cut benefit of CP in hospitalized patients with severe COVID-19, other studies have shown that if given early in the course of the disease, it might be a useful therapeutic option. In this retrospective study, we demonstrated that early treatment (within 3 days of hospitalization) was significantly associated with reduced 28-day mortality compared with those patients treated beyond day 3. The results from our study add up to the scientific evidence on the use of CP as a relatively safe, cheap, and possibly effective therapy in certain patients suffering from severe SARS-CoV-2 infection.

## INTRODUCTION

The relevance of the humoral immune response in viral infections is a well-known phenomenon exemplified in many clinical settings. For instance, studies carried out several decades ago in Argentina provided evidence of the benefit of convalescent plasma (CP) transfusion in patients with Argentine hemorrhagic fever (AHF) caused by the Junín virus. Passive immunotherapy with intravenously immune plasma from AHF convalescents led to significantly reduced mortality (1), more evident when employing plasmas containing higher levels of specific neutralizing antibodies (2). Extending these findings, studies in patients with SARS-CoV-1 revealed that they were discharged earlier when given CP (3).

Within the context of the current COVID-19 pandemic, there are many studies on the use of CP in patients with COVID-19 that in general seem to point out a possible benefit (3, 4). In our country, a randomized, double-blind, placebo-controlled study was carried out including patients with severe pneumonia with a median from the onset of symptoms of 8 days (5). This study failed to demonstrate differences in the disease course of the patients on day 28, after randomizing 228 patients to receive CP (with a median anti-SARS-CoV-2 titer of 1:3,200) or 105 patients to receive placebo (5). A further study assessing the activity of CP when administered during early infection in older adults revealed, however, that this intervention was able to confer statistically significant protection against the progression of COVID-19 disease (6). A more recent randomized controlled trial (RCT) indicates that early administration of CP as an outpatient strategy showed a relative risk reduction in the hospitalization rate of 54% (7). A retrospective study and a systematic review analyzing the use of CP among hospitalized patients with COVID-19 not requiring mechanical reported that the benefit of its use was most apparent when receiving CP with high anti-SARS-CoV-2 IgG antibodies and earlier in the course of the disease (8, 9). It should be noted that no beneficial effect of CP was shown when administered to patients receiving invasive mechanical ventilation (IMV) (10).

Shortly after the scourge of COVID-19 in the province of Santa Fe, Argentina, the Ministry of Health recommended the use of CP transfusion obtained from patients who recovered from the disease. Outcomes of hospitalized patients with COVID-19 disease who were treated with CP between June and October 2020 are analyzed in the present report, aiming to assess potential factors that could be associated with clinical outcomes such as baseline conditions, timing of CP administration, and levels of specific anti-spike (S) antibodies present in CP.

## MATERIALS AND METHODS

### Study design

This is a retrospective study analyzing hospitalized patients with COVID-19 disease residing in the Province of Santa Fe, Argentina, who received one or two units of CP according to the official protocol between June and October 2020. All plasma donors were screened for transfusion-transmitted infections (HIV, HBV, HCV, Chagas disease, syphilis, brucellosis, and HTLV I/II) and recipients were monitored for events such as allergic reaction, anaphylaxis, non-hemolytic febrile reaction, transfusion-related acute lung injury, and transfusion-associated cardiac overload.

Patients were eligible to receive CP if they were older than 18 years of age, had a confirmed diagnosis of SARS-CoV-2 infection by real-time polymerase chain reaction (RT-PCR) from a nasopharyngeal swab or respiratory secretion sample, and were hospitalized with a moderate or severe COVID-19 disease, and had the informed consent form signed by the patient or legal representative. On the other hand, patients were excluded if they had a prior history of moderate to severe adverse reactions to blood components, were pregnant, had other severe concomitant infections, had active intracranial bleeding, or had an acute myocardial infarction.

### Definitions

Those patients with evidence of lower respiratory disease but with an oxygen saturation by pulse oximetry (SpO2) above 93% breathing at room air were considered to have a moderate COVID-19 disease; while severe COVID-19 disease was defined as those individuals having any of the following: SpO2 ≤ 93%, respiratory rate ≥ 30/min, the ratio of arterial partial pressure of oxygen to fraction of inspired oxygen (PaO_2_/FiO_2_) <300 mmHg, progression of pulmonary infiltrates > 50% within 24 to 48 hours, or impaired consciousness (11). Transfusion-related adverse events, including febrile and anaphylactic reactions, allergic skin and/or pulmonary symptoms, hypotension, or tachycardia, were recorded by each investigator up to 7 days after CP administration.

### Clinical outcome

All-cause mortality at day 28 was the primary outcome analyzed and variables associated with this outcome were explored. The clinical status 28 days after receiving the intervention based on the proportions of patients in each stage of a simplified World Health Organization (WHO) clinical progression scale was also studied (12). This scale includes five ordinal categories: (i) discharged, (ii) hospitalized without supplemental oxygen requirement, (iii) hospitalized with supplemental oxygen requirement, (iv) need for IMV or extracorporeal membrane oxygenation (ECMO) or vasopressors, and (v) death. The rate of reported adverse events associated with CP transfusions was also assessed.

### CP administration protocol

ABO typing was performed on potential candidates for the study and the CP unit could be administered regardless of group compatibility if the anti-A and/or anti-B antibodies had a titer no greater than 1/64 (as per national regulations). The 200–250 mL of the prepared CP was infused at a rate of 100–200 mL/hour, based on the patient’s hemodynamic conditions, and supervised by a Transfusion Medicine specialist. The attending physician could prescribe a second dose of CP 48 hours later if considered necessary. Criteria for discontinuing CP comprised the following: cutaneous or mucosal manifestations, a significant decrease in the systolic blood pressure, tachycardia with resting heart rate greater than 130 bpm, or bradycardia less than 40 bpm, and acute onset of gastrointestinal or respiratory symptoms. Concomitant therapies for the treatment of SARS-CoV-2 infection, such as corticosteroids, azithromycin, remdesivir, and tocilizumab, among others, were allowed.

#### Selection of CP

Donors of CP were required to meet all of the following: documented evidence of COVID-19 (positive RT-PCR or the presence of specific antibodies for SARS-CoV-2), being free of symptoms for at least 28 days before donation, and no history of pregnancy/abortion (or absence of anti-HLA antibodies). ABO, Rh, and Kell blood group systems were studied, in addition to Irregular Antibody Tests (IAT). The presence of total anti-SARS-CoV-2 antibodies was investigated in all the donors by the electrochemiluminescence method. Elecsys anti-SARS-CoV-2 test (Roche) was the automated immunoassay used for the *in vitro* qualitative detection of antibodies anti-N (including IgG) to SARS-CoV-2 in most of the plasma recovered from donors. The assay uses a recombinant protein representing the nucleocapsid (N) antigen in a double-antigen sandwich assay format, which favors the detection of high-affinity antibodies against SARS-CoV-2. Since these antibodies have been positively correlated with neutralizing antibodies in the neutralization assay (13), a cut-off index result greater than 10 was applied for CP selection. A comparison of the mortality rate at day 28 was performed between those receiving CP according to the revised antibody titers as recommended by the Food and Drug Administration (FDA) (≥210 units/mL) for this assay (14).

### Statistical analysis

Comparisons among groups were made according to the potential predictors. Categorical variables were analyzed by the χ^2^ or Fisher’s exact test when applicable, whereas the analysis of variance and Student’s *t*-test were used to evaluate differences in mean values for quantitative symmetric variables. Otherwise, non-parametric analyses were applied. To assess the eventual effects of other variables on the means of quantitative outcomes, general linear models were applied. This procedure provides regression analysis and analysis of variance for one dependent quantitative variable adjusted by one or more factors. The timing of plasma administration was categorized as less than 3 days (reference category), between 3 and 5 days, and beyond 6 days, after hospitalization.

To identify independent predictors of 28-day mortality, variables differing between survivors and non-survivors with a *P* value < 0.10 were entered into an unconditional multivariable logistic regression model, using a forward stepwise analysis. Unadjusted and adjusted risks were expressed as odd ratios (OR) and 95% confidence intervals (95% CI). For all analyses, two-tailed *P* values < 0.05 were considered significant. Data were analyzed with STATA 9.0 software.

## RESULTS

Between 1st June and 31st October 2020, 790 hospitalized patients with COVID-19 disease received at least one CP infusion. For this study, those who received CP while on IMV (*n* = 310) were withdrawn from the analysis, leaving 480 treated patients. Of these patients, 361 (75.2%) were in the general ward and 119 (24.8%) in the intensive care unit (ICU) at the time of CP administration. The median age of this cohort of patients was 60 years (interquartile range: 49–69 years), and 320 (66.7%) were males (Table 1). The vast majority of the patients (98.7%) were receiving supplemental oxygen when CP was infused. Only 19.7% (*n* = 91) of the patients did not have any comorbidity, and the most frequent ones were hypertension, obesity, cardiovascular disease, diabetes, and chronic obstructive pulmonary disease (COPD)/asthma. Most of the patients (98%) received corticosteroids, while few were treated with azithromycin or clarithromycin (13%), oseltamivir (12%), hydroxychloroquine (0.2%), and remdesivir (0.1%). The SpO2 was below 93% in 89.3% of the patients and 93.75% (*n* = 450) and 6.25% (*n* = 30) received one CP and two CP infusions, respectively. By day 4 and day 7 of hospitalization, 82.1% and 95.6% of the patients had received the CP infusion, respectively.

**TABLE 1:** Characteristics of 480 patients treated with CP^*a*^

	Median (IQR)	Range
Age	60 (49–69)	24–95
Days between symptoms initiation and hospital admission	6 (3–9)	0–20
Days between symptoms initiation and CP transfusion	8 (6–11)	1–31
Days between hospital admission and CP infusion	2 (1–4)	0–17
Respiratory rate	24 (20–28)	15–45
Oxygen saturation (%)	90 (88–91)	65–99
Pa02/FiO2 (*n* = 411)	190 (150–230)	15–470
Anti-S IgG levels (IU/ml in the CP, quantitative *n* = 389)	130 (54.4–250)	0.4–250
COI anti-SARS-CoV2 total antibodies (qualitative, *n* = 469)	66 (24–121)	0.4–250
**Categorical data**	**n**	**%**
Male sex	318	66.4
Number of comorbidities		
0	110	23.0
1	148	30.9
2	112	23.4
3	64	13.4
Four or more	45	9.4
Most frequent comorbidities	**n**	**%**
Hypertension	244	55.1
Obesity	171	36.2
Cardiovascular disease	123	25.6
Diabetes	137	31.6
COPD/asthma	102	21.5
Simplified WHO scale baseline category:	**n**	**%**
2- Hospitalized without oxygen need	6	1.3
3- Hospitalized with an oxygen requirement	474	98.7

^*a*^ COPD: chronic obstructive pulmonary disease; CP: convalescent plasma; COI: cutoff index

At 28 days of follow-up, 250 patients were discharged (52.1%), 131 (27.3%) remained hospitalized without supplemental oxygen requirement, 16 (3.3%) remained hospitalized with oxygen requirement, 27 (5.6%) were on IMV, and 56 (11.7%) had died.

**Fig 1:**
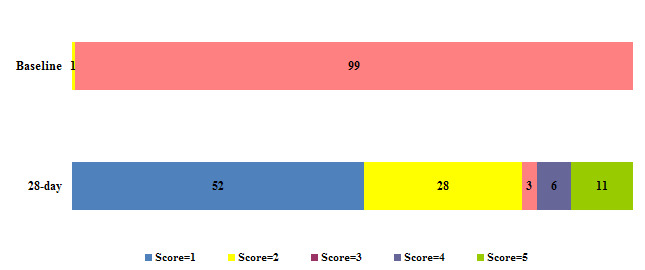
Proportion of patients in each of the simplified WHO scores at baseline and at day 28 (*n* = 480). Score 1: discharged; score 2: hospitalized without supplemental oxygen; score 3: hospitalized with supplemental oxygen; score 4: invasive mechanical ventilation, ECMO or vasopressors; score 5: death.

None of the 6 patients that, at baseline, were hospitalized without oxygen requirement and 56 (11.8%) of those who were hospitalized with oxygen (*n* = 474), died through 28 days of hospitalization (Fig. 1).

### Comparisons between survivors and non-survivors at day 28 post-CP

The univariate regression analysis of non-survival at day 28 post-infusion of CP found no association with sex, ABO blood type, COPD/asthma, obesity, hypertension, or diabetes (Table 2). However, the chance of dying increased by 6% and by 26% for each 1-year age increase and each comorbidity added, respectively. Also, the risk of dying was almost two times higher in patients who were directly admitted to the ICU versus those in the General Ward. Interestingly, for each day of delay in the CP administration, the risk of death increased by 32%. Indeed, those patients who received CP after 3 days of hospitalization had a fourfold higher chance of death when compared with those treated with CP before the second day of hospitalization (95% CI = 2.1–7.6; *P*= <0.01). Qualitative and quantitative determinations of antibodies against SARS-CoV-2 were not associated with 28-day mortality in this analysis.

**TABLE 2:** Univariate logistic regression analysis for 28-day mortality after CP infusion^*a*^

Variables	Odds ratio	*P*	95% CI
Male sex	1.17	0.615	0.64–2.14
ABO blood type A	1.11	0.726	0.62–2.00
COPD/asthma	1.40	0.317	0.73–1.27
Obesity	0.62	0.145	0.33–1.48
Hypertension	1.44	0.253	0.77–2.71
Diabetes	1.35	0.350	0.72–2.51
Cardiovascular disease	2.39	0.005	1.30–4.38
Age (continuous variable)	1.06	<0.001	1.03–1.08
Age >60 versus ≤60 years	2.69	0.001	1.48–4.91
Number of comorbidities	1.26	0.054	1.00–1.60
ICU admission	1.82	0.046	1.01–3.29
Invasive mechanical ventilation use	17.5	<0.001	5.08–60.28
Days until CP infusion (as a discrete variable)	1.32	<0.001	1.76–1.48
Days until CP infusion, >3 versus ≤3	3.95	<0.001	2.05–7.60
Qualitative antibodies (continuous variable)	1.00	0.860	0.99–1.01
Qualitative antibodies < median	1.39	0.280	0.76–2.54
Quantitative antibodies (continuous variable)	0.99	0.077	0.99–1.00
Quantitative antibodies < 210 IU/mL	1.49	0.260	0.74–2.03

^*a*^ COPD: chronic obstructive pulmonary disease; ICU: intensive care unit; CP: convalescent 427 plasma.

A quantitative analysis of the transfused CP based on the FDA recommendations (antibody levels of at least 210 units/mL using the Elecsys Anti-SARS-CoV-2 S assay) was performed in 389 (81%) of the 480 CP units administered to the patients as the first CP infusion (excluding the second dose of the 30 patients treated with two doses of CP) resulting in a median value of 131 units/mL. The proportion of transfused CP with antibody titers above (9.0%) or below (12.9%) 210 units/mL was similar among subjects that died on day 28 (*n* = 56) (*P* = 0.258).

**Fig 2:**
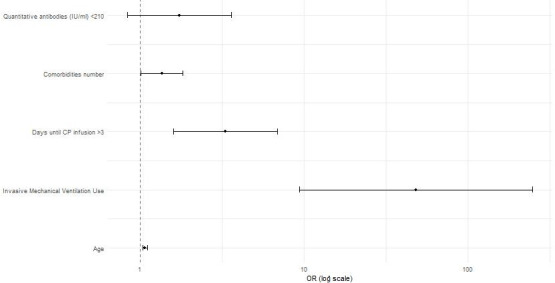
Odds ratio (logarithmic scale) of factors associated with mortality at day 28 after CP administration.

Factors contributing to fatal outcomes during the 28 days of hospitalization were analyzed by multivariate logistic regression analysis, as shown in Table 3 and Fig. 2. Upon adjusting for possible confounding factors, the risk of death was 48 times higher in patients requiring IMV and threefold higher in patients who received CP after the third day of hospitalization compared to those treated with CP before day 3. Likewise, the risk of dying increased by 36% and 7%, for the addition of each comorbidity and every 1-year age increase, respectively.

**TABLE 3:** Multivariate logistic regression analysis on factors associated with mortality throughout 28 days after CP^*a*^

Variables	Odds ratio	*P*	95% CI
Age	1.07	<0.001	1.04–1.11
Invasive mechanical ventilation needs	48.14	<0.001	9.36–247.55
Days until CP infusion, >3 versus ≤3	3.32	0.001	1.60–6.91
Comorbidities number	1.36	0.042	1.01–1.83
Quantitative antibodies, <210 versus ≥210 (IU/mL)	1.74	0.134	0.84–3.60

^*a*^ CP: convalescent plasma. Pseudo-R^2^ = 0.2414 (indicating good model fit).

The analysis of quantitative CP antibodies as a categorical variable (less than 210 IU/mL versus more than 210 IU/mL) showed no significant association with mortality (adjusted OR = 1.744; *P* = 0.134; 95% CI = 0.843–3.609).

### Safety of CP administration

Among all CP infusions, only three patients (0.6%) developed transfusion-related reactions, which included mild allergic reactions, an elevation of liver transaminases, and increased oxygen requirement (room air O_2_ saturation decreased from 92% the day of CP administration to 88% the following day, with improving values the following days).

## DISCUSSION

To cope with infectious agents, T and B lymphocytes are endowed with a great capacity to recognize foreign antigens. One activated T cells proliferate and circulate to protect the host by destroying infected cells in addition to activating B cells for antibody production. Antibodies bind to the pathogen, or its products (15, 16). In the case of viruses, that is, SARS-CoV-2, antibodies can inhibit virus attachment to specific host cell receptors, block the uncoating of the virus, and therefore interfere with productive infection. In this way, viral neutralization constitutes a major critical event involved in antibody-mediated protection against viruses (13, 15, 16).

This retrospective study found that, among hospitalized patients with severe COVID-19 pneumonia treated with CP, its early administration seems to be associated with decreased mortality. In the multivariate logistic regression analysis, the administration of CP after day 3 versus those treated before day 3 of admission was one the most important factors associated with 28-day mortality. Along with this finding, a retrospective study of 341 hospitalized patients with COVID-19 treated with high anti-S receptor binding domain antibodies CP matched with a control group that did not receive CP found a significant 60-day mortality decrease in those treated with CP if given within 3 days of hospitalization (17). However, another group of researchers could not find any difference in the inpatient mortality between a group of patients treated with CP within 3 days of admission and a propensity score matched control (18). More recently, a meta-analysis including 30 RCTs showed some level of efficacy of CP provided there was a higher neutralizing titer in the transfused plasma and a shorter time to randomization (19). Current guidelines recognize that high-titer CP may be more effective if given early in the course of hospitalization and in immunocompromised patients, particularly if they lack (or have low titers of) baseline anti-SARS-CoV-2 antibodies, and that additional trials are needed (20). The demonstration that the earlier the CP the better its benefit may be taken to imply a faster clearance of viral burden. In relation to this, passive immunotherapies may be more beneficial in patients lacking specific anti-SAR-CoV-2 antibodies, as may be the case in patients transfused at the beginning of the clinical disease. In the recovery trial (21), those patients lacking specific antibodies at baseline had significantly higher 28-day mortality (30%) than those with antibodies. In the same study, the administration of monoclonal antibodies combination decreased the mortality compared to placebo (24% versus 30%) only in patients without specific antibodies at baseline (21).

As it has been shown (22), the mortality observed in this cohort of patients was associated with age and the number of comorbidities. Even the beneficial impact of using CP with high anti-S titers has been demonstrated by others (8, 17), with the methods utilized in our series of patients, we were unable to detect any clinical influence related to the quantity of anti-SARS-CoV-2 antibodies present in the transfused CP. Even though the overall titer of anti-S antibodies does not necessarily reflect their neutralizing activity, the lack of effect associated with the antibody levels may reinforce the view of an increased chance of CP beneficial effect provided the plasma is administered shortly after the clinical diagnosis of COVID-19.

The results from this research confirm the safety profile of the CP transfusion as only 0.6% of the patients suffered from an infusion-related reaction and none of the CP infusions had to be interrupted.

The main limitations of this study include (i) the observational nonrandomized study design that led to the CP administration to a remarkably diverse population in terms of disease severity, days since admission or diagnosis, etc., and (ii) the inability to directly measure neutralizing antibodies present in the transfused CP, although a correlation between the electrochemiluminescence anti-SARS-CoV-2 method with neutralizing antibodies has been shown (13). Beyond such constraints, results from this study point out that for patients with severe COVID-19 pneumonia, the early administration of CP confers a better chance of decreased fatal outcomes.
